# Snowboarders’ Knowledge of the FIS Rules for Conduct on Ski Slopes

**DOI:** 10.3390/ijerph17010316

**Published:** 2020-01-02

**Authors:** Luis Carus, Isabel Castillo

**Affiliations:** 1 Faculty of Health and Sport Sciences, University of Zaragoza, 22002 Huesca, Spain; 2 Faculty of Business and Public Management, University of Zaragoza, 22002 Huesca, Spain; icastillo@unizar.es

**Keywords:** accident prevention, snowboard, level of knowledge, FIS Rules for Conduct on ski slopes

## Abstract

The objective of the present study is to assess snowboarders’ general perceptions of safety and knowledge of existing rules and both active and passive knowledge of the International Ski Federation (FIS) regulations in order to contribute to defining target groups for specific educational interventions in the field of injury prevention. Data were drawn from random interviews conducted with 918 snowboarders during the 2017–2018 winter season at five ski resorts located in the Spanish Pyrenees. To collect the data, a questionnaire assessing personal characteristics (gender, age, origin, and self-reported skill), general perception of safety, general request for rules, and knowledge of existing rules was used. Pearson’s Chi-squared tests were performed to compare characteristics between groups. The study revealed, for accident prevention purposes, a concerning lack of general knowledge of existing rules. Risk-inducing situations that could result in severe injuries were largely assessed incorrectly. The appropriate intuitive behavior increases with age and experience: youths and beginners are less able to implement the FIS rules than older and more experienced snowboarders. Stakeholders, such as parents, ski resorts, clubs or schools, should direct educational efforts at high-risk groups. Further research is needed to determine the causal relation between snowboard-related injuries and disregard of FIS rules.

## 1. Introduction

Severe and life-threatening injuries among participants in snow sports have increased [1]. What makes primary prevention, including analysis of the magnitude of the problem, identification of risk factors, and promotion of safety behavior, an essential area of concern for all parties involved (enthusiasts, ski resorts, physicians, researchers, etc.).

Previous research has shown that a lack of knowledge of safety rules prevails among injured skiers rather than among non-injured [2]. Alpine skiing and snowboarding on traditional slopes are associated with a risk of injury, while skiers and snowboarders’ risk of injury is highly dependent on their knowledge of proper behavior on the slopes [2,3,4]. Therefore, since inappropriate and hazardous behavior may be intentional or a consequence of a lack of knowledge, the regulation of ski and snowboard slope activities is essential in informing users in order to reduce accident, injury, and severity rates [5]. 

With the aim of promoting safe skiing on traditional slopes, as early as 1967 the International Ski Federation (FIS) introduced rules that applied to skiers [6], and later on, with the consolidation of snowboarding as an alpine discipline in its own right, updated them in order to embrace the enthusiasts of the new technique [7].

On-slope skiers and snowboarders are expected to be familiar with these rules prior to practicing their sports [7]. Furthermore, court decisions on ski and snowboard accidents have consistently been based on the behavior required by the FIS Rules of Conduct for skiers and snowboarders while assessing the conduct of the parties. For example, building on these regulations, the courts found defendants guilty when plaintiffs were able to prove that they had voluntarily and recklessly skied at excessive speed in places with no visibility (rules 1 and 2), in areas particularly signaled to be passed at a very low speed (rules 1, 2, and 8), or with disregard for other skiers who had paused at the sides of the ski runs or were skiing at clearly lower levels of the slope (rules 3 and 4) [8]. 

Few studies have evaluated the effectiveness of ski and snowboard instruction in increasing the share of safer behaviors and reducing the risk of injury [9,10,11], and only one work by Hildebrandt et al. investigated skiers’ knowledge of existing rules [5]. However, the authors acknowledge that since the FIS rules also apply to snowboarders, further research is required in order to examine the knowledge of the latter and, through well founded comparison, investigate modality-related behavior and differences between groups.

In order to fill this gap, this study investigates snowboarders’ general perception of safety on traditional slopes and their knowledge of the existence of rules, and both active and passive—intuitive behavior in a given situation on the slopes—actual knowledge of FIS regulations. It also discusses its findings against those obtained by Hildebrandt et al. for regular slopes skiers [5]. These data, in correlation with snowboarders’ gender, age, self-reported skill level, and origin, may contribute to defining the target groups for specific education interventions. 

## 2. Materials and Methods

According to the Spanish Ski and Mountain Resorts Association (ATUDEM), 3,717,433 people visited the 18 ski resorts located in the Spanish Pyrenees during the 2017–2018 winter season, of which around 35% were snowboarders (~1,301,000) [12]. Our research data were collected through a survey performed between December 2017 and April 2018 in different weather conditions and at different times of the day, at five ski resorts located in the Spanish Pyrenees, including the largest one in the mountain range, with information provided by a largely representative random sample of 918 users.

The participants were approached according to sets of daily computer-generated random numbers, and informed consent was obtained from them, except for minors whose consent was sought from their parents who had to be present. In cases where this requirement could not be met the next available minor was approached.

The survey was conducted by experienced interviewers who had received a specific instruction for the task on matters such as the point where to approach the interviewees (e.g., resort parking lots, hospitality and catering facilities), how to make them comfortable (e.g., their faces to the sun or backs to the wind) or how to conduct the interview safely (e.g., places not to be stopped at).

In order to not only obtain data on snowboarders’ perceptions of safety and knowledge of existing rules, but also to make them comparable to those obtained from skiers by Hildebrandt et al. [5], we used the questionnaire previously devised and validated by the authors to assess personal data (gender, age, origin (“local (Pyrenean citizen)/non-local (other)”), and self-reported skill level (“beginner/intermediate/expert”)), general perceptions of safety (“How safe do you feel on the ski slopes?”), perceived importance of rules (“Are rules important on the ski slopes?”), general knowledge of existing rules (“Do specific rules for the ski slopes exist?”/”Can you provide the name for these rules?”), and actual, active and passive—intuitive behavior for given situations on the ski slopes (Table 1)—knowledge of the FIS rules. 

Except for the open question regarding actual active knowledge of existing rules devised to assess those the participants knew (“Can you please mention the FIS rules you know?”), questions were closed, with “very safe/safe/unsafe/very unsafe” as possible choices offered to participants to assess responses on perceptions of safety; “important/unimportant” as possible choices to assess responses on perceived importance of rules; and correct (“yes”) or incorrect (“no or I don’t know”) as possible choices to assess responses on questions related to general knowledge of FIS rules and those related to passive knowledge. Only those participants who acknowledged knowing the existence of general rules for ski slopes were asked about their ability to provide the proper name of these rules and to mention the existing rules they knew.

Tables were used to display descriptive analyses. They included both items, expressed as percentages, and results of Pearson’s Chi-squared tests to assess independence between groups within gender, age, origin, and skill level. The results were considered statistically significant at *p* < 0.05 level. Data were analyzed using Minitab 19 (State College, PA, USA) for Windows. This study was conducted following the approval of the University of Zaragoza research board. The research was carried out following the rules of the Declaration of Helsinki, regarding research involving human subjects (revised in 2013 in Fortaleza, Brazil). 

## 3. Results

A total of 918 snowboarders out of 942 agreed to complete the questionnaire (97.5%). Table 2 presents the demographic profile of the sample: more than a half of the respondents were males, between 25 and 59 years old, non-local snowboarders, and self-reported an intermediate skill level.

As shown in Table 3, most of the snowboarders felt safe (55%) or very safe (31%) while riding on the resort slopes. A limited number of them acknowledged feeling unsafe, while a mere 2% felt very unsafe. Significantly, seniors felt less safe than adults and youths, and the latter particularly safe (*p* < 0.001). At the same time experts felt safer than intermediates and beginners (*p* = 0.047). Gender and residency of the participants did not have a significant effect on their sense of safety.

As many as 68% of respondents thought rules to be important (Table 4). There was a significant difference in the responses between all ages: adults and seniors were more likely to agree that rules were necessary, whereas youths were more likely to disagree (*p* < 0.001). The difference between levels of skill was also significant, and experts were the most likely to perceive rules as important (*p* = 0.003). Referring to gender and origin the results showed no significant differences.

Differences regarding the knowledge of the existence of general rules and the ability of snowboarders to provide the correct name of the rules are shown in Table 5. In total, 62% of respondents were aware that general rules existed, and 29% of them (18% of the sample) could provide the proper name for these rules. Age had a significant bearing on the knowledge of the existence of general rules. Only 37% of the youngest participants knew about the existence of rules against 82% of adults. Origin and skill level were also significant factors when it comes to knowledge of existing rules. Remarkably, beginners revealed a large deficit in knowledge (20%), whereas local snowboarders displayed the highest level of knowledge (84%). The term FIS rules was more familiar to seniors and to the most skilled snowboarders, while youths, beginners, and intermediates were hardly able to identify it.

Among those who knew that rules for general application on slopes existed, regardless of whether they knew their name (FIS Rules) or not, the most common responses to the open question “Can you mention the existing rules you know?” (Table 6 and Table 7) were the second (“Control of speed when skiing or snowboarding”) and the ninth (“Assistance”) rules. Between forty and sixty percent of them mentioned rules number three, four, and eight. The first (“Respect for others”) and fifth (“Entering, starting and moving upwards”) rules were scarcely mentioned (~10%), while the sixth (“Stopping on the piste”), seventh (“Climbing and descending on foot”) and tenth (“Identification”) were rarely identified. The influence of age proved to be significant in reference to the mentioning of half of the rules, specifically, rules 1, 2, 5, 8 and 9, showing seniors as more accurate than adults and youths. So did the influence of skill level, proving experts to be much more accurate than beginners and intermediates, when acknowledging rules 1 to 5, 8 and 9. Origin had a significant impact when participants quoted rules 1, 3, 4 and 9: locals showed more knowledge than non-locals (rules 1 and 4) or vice versa (rules 3 and 9). Gender had no significant influence, except for rule 4, which was much more familiar to males than females.

Participants were asked twelve close-ended questions intended to apprehend snowboarders’ passive knowledge of the FIS rules through evaluating their alleged behavior in given situations on the slopes (Table 8 and Table 9). The second (“Ride in control of speed”), fifth (“Entering, starting and moving upwards”), eighth (“Respect for signs and markings”), and ninth (“Assistance”) questions were answered correctly at a rate above 90%, though only the latter exceeded 95%. Questions related to the “Choice of route” (questions 2, 3, and 4) and to “Overtaking” (question 5) were answered incorrectly more often. In this sense, around 25% of the participants thought that a faster rider as well as the one approaching from the right had priority while the person in front did not, and 30% did not know that overtaking was possible to the right, left, above, and below others. Questions related to the sixth rule (“Stopping on the slope”) and the seventh (“Climbing and descending on foot”) were less frequently answered correctly. In total, 50% of the participants thought it was correct to stop anywhere on the slope and 60% did not know that climbing and descending on foot required keeping to the side of the slope.

The most outstanding lack of knowledge was found with respect to the tenth rule (“Identification”). In total, 62% of snowboarders did not think it was necessary for a responsible party or a witness to exchange personal information following an accident. The role of age was found significant in reference to the correction of responses to almost every question, but to questions 1 and 11, mainly seniors and adults were the groups who gave the highest number of correct answers. Skill level proved to be significant in every question but the eleventh, showing the group of experts to always have the highest percentages of correctness. Origin had more impact for questions 1, 6, and 7 (non-locals over locals), and 4 (locals over non-locals). Gender did not have a significant influence, except for question 12 (males over females).

## 4. Discussion

Previous research has shown that alpine snow sports are associated with a risk of injury and that the risk of injury for skiers and snowboarders is highly dependent on their knowledge of the proper behavior on the slopes [2,3,4]. An important way of reducing accidents and thus injuries is to ensure that they are sufficiently acquainted with the FIS Rules for Conduct on slopes [5]. 

To the best of our knowledge this study is the first to assess the snowboarders’ perceptions of safety, general knowledge of existing rules, and actual, active, and passive understanding of the FIS rules with regard to gender, age, origin, and skill level, and to discuss its findings against those previously obtained for regular slopes skiers [5].

Our results revealed that 14% of snowboarders feel unsafe, mainly seniors and beginners. It has been suggested that communication of FIS rules in the media, at free informative initiatives in snow regions and on appealing banners displaying the FIS rules present at the entrance to lifts and cable cars [5], or other premises frequented by skiers and snowboarders, might reduce accidents consequently improving the feeling of safety on the slopes.

This study has also found that young snowboarders in particular, in contrast to adults and seniors, tend to discard rules. In this respect, Ruedl et al. stated that risk-taking behavior on the slopes is associated with youths [13], and Brooks et al. revealed that the youngest snowboarders were at the highest risk of injury [14]. It seems that a special educational effort should be made to improve their safety-seeking behaviors, and for that purpose the Canadian Pediatric Society recommends that physicians should provide office-based anticipatory guidance, counseling families to become familiar with and adhere to the Alpine Responsibility Code [15].

A total of 62% of snowboarders knew about the existence of rules for general application on slopes but, out of the participants who knew that rules existed, only 29% were aware that the rules were called FIS rules. Young snowboarders and beginners turned out to be the groups that showed the poorest levels of awareness of the existence of rules, in contrast to the highest levels of awareness among locals and experts. Our results are consistent with those of Macnab et al. who found that 25% of young injured skiers and snowboarders had never heard of the rules [2]. Among the snowboarders who knew that rules existed experts were significantly familiar with the term FIS rules and seniors the most familiar with it. The relative modernity of snowboarding could partly account for this remarkably low acquaintance of snowboarders with the FIS rules. However, the stakeholders involved in conveying the latter to snowboarders, since snowboarding was included in the FIS regulations in 2002 (FIS, resorts, snowboard schools, clubs, etc.), might need to reappraise the effectiveness of their present communication policies for reaching the so far misinformed snowboarders.

The active knowledge of general rules and the intuitive behavior of snowboarders on the slopes seem to align. Rules such as the second (“Control of speed when skiing or snowboarding”) and the ninth (“Assistance”) were the most commonly quoted and the questions related to them (1 and 11) also registered the highest rates of responses. The like happens with less acknowledged rules, with the only exception of rules 5 (“Entering, starting and moving upwards”) and 8 (“Respect for signs and markings”) that though infrequently quoted, the intuitive behaviors related to them were frequently assessed correctly. However, it is especially worrying that a high percentage of risk-inducing situations that might result in collisions between slope users (the most common cause of accidents often resulting in severe injuries and leading to legal action [8]), such as stopping anywhere or climbing and descending on foot without keeping to the side of the slope, were assessed incorrectly.

In comparison with skiers, the demographic profile obtained from the data on snowboarders present some substantial differences, mainly in gender and age: the ratio of males over females is higher (68% vs. 55%), as is the ratio of youths over adults and seniors (40% vs. 25%). It is noticeable that young snowboarders tend to discard rules in a proportion that more than doubles that of young skiers (59% vs. 27%) [5]. Snowboarders’ knowledge about the existence of rules for general application on slopes is below that of skiers (62% vs. 75%) and, out of the participants who knew that rules existed, the percentage of them that were aware that the rules were called FIS rules, was much lower than that previously found for skiers (29% vs. 45%) [5]. These differences of knowledge and behavior between skiers and snowboarders might have a sociological and/or psychological explanation that escapes the objective of this study, but would be worthwhile investigating.

Despite these differences, the actual knowledge of rules and the appropriateness of intuitive behavior mainly change along with age and experience of both snowboarders and skiers. Young and unexperienced snowboarders and skiers showed the largest gap in active knowledge of rules and were less able to implement intuitively the FIS rules. It seems highly advisable that these groups should attend classes in which educators ensure their knowledge of the rules. Up to now, the FIS rules are not legally enforced and mostly apply in court cases and for insurance purposes. However, the aim should be to treat the rules as preventative measures. They comprehend all aspects of safe skiing and snowboarding, and active education measures are advisable in order to improve the awareness of such regulation. A variety of individuals and institutions, such as parents, ski schools, resorts or clubs and different strategies should be involved in the implementation of such measures. Deeper research should help to choose the most effective policies for this purpose.

The study findings are limited because the data were obtained from snowboarders surveyed at resorts located in the same mountain range in one country and may not apply to others. Although the results may be considered useful to generate new hypotheses on snowboarders’ knowledge of the FIS rules, further research is needed to investigate causal relationships between snowboarders’ injuries and disrespect for rules. However, this research would furnish stakeholders and particularly ski resort risk managers with valuable information to assess the dimensions of the problems to be avoided or solved through a wide range of possible information and safety policies.

## 5. Conclusions

We found that only a little proportion of the participants in this study was aware that rules for general application on ski slopes were called FIS rules. The appropriate general knowledge of the FIS rules and the intuitive behavior in given situations seem to align, but the lack of knowledge of key rules, related to most and more severe accidents occurring on ski slopes, must be urgently addressed. These findings suggest that effective strategies for the communication of the FIS Rules for Conduct on ski slopes must be implemented, focusing on younger and more unexperienced snowboarders as the main target.

## Figures and Tables

**Table 1:** Passive knowledge: intuitive behavior for given situations on ski slopes (R: corresponding FIS rule).

Q1. I have to ride with care and in control (R2). Q2. The slower person is responsible to ensure enough space for the faster one (R3). Q3. The person from the right has priority (R3). Q4. The person in front has priority (R3). Q5. I can overtake above, below, to the right and to the left (R4). Q6. When entering and starting, I have to look up and down the slope (R5). Q7. I have priority when moving upwards (5). Q8. I can stop everywhere in the slope (R6). Q9. Climbing and descending on foot must kept to the side of the slope (R7). Q10. I need to observe signs showing warnings of dangers (R8). Q11. I have to render first aid (R9). Q12. It is a duty to provide personal information as a witness (R10).

**Table 2:** Respondents profile.

Profile	*n*	%
Gender		
Male	626	68
Female	292	32
Age		
Youths (14–24)	371	40
Adults (25–59)	493	54
Seniors (≥60)	54	6
Origin		
Local	383	42
Non-local	535	58
Skill level		
Beginner	101	11
Intermediate	515	56
Expert	302	33

**Table 3:** Perception of safety on the slopes.

Safety	Very Safe	Safe	Unsafe	Very Unsafe	*p*
Gender					0.823
Male	32	55	12	1	
Female	29	32	14	3	
Age					<0.001
Youths (14–24)	54	40	5	1	
Adults (25–59)	17	67	14	2	
Seniors (≥60)	4	37	54	6	
Origin					0.919
Local	32	55	12	2	
Non-local	31	58	13	2	
Skill level					0.047
Beginner	17	11	38	2	
Intermediate	27	56	13	1	
Expert	42	52	3	3	
Total	31	55	13	2	

Data in %.

**Table 4:** Perceived importance of rules.

Importance	Important	Unimportant	*p*
Gender			0.497
Male	67	33	
Female	70	30	
Age			<0.001
Youths (14–24)	41	59	
Adults (25–59)	86	14	
Seniors (≥60)	96	4	
Origin			0.561
Local	67	33	
Non-local	69	31	
Skill level			0.003
Beginner	58	42	
Intermediate	68	32	
Expert	72	28	
Total	68	32	

Data in %.

**Table 5:** General knowledge of International Ski Federation (FIS) rules.

Knowledge	* Rules Exist	*p*	** Named FIS-R	*p*
Gender		0.498		0.318
Male	61		33	
Female	64		30	
Age		0.019		<0.001
Youths (14–24)	37		59	
Adults (25–59)	82		15	
Seniors (≥60)	46		4	
Origin		<0.001		0.593
Local	84		33	
Non-local	46		31	
Skill level		<0.001		<0.003
Beginner	20		42	
Intermediate	56		32	
Expert	85		28	
Total	62		32	

* Correct answers: *n* (%). ** Correct answers of those who knew that rules for general application exist: *n* (%).

**Table 6:** Active knowledge of existing rules (*).

FIS Rules	R1	*p*	R2	*p*	R3	*p*	R4	*p*	R5	*p*
Gender		0.398		0.718		0.639		0.021		0.253
Male	9		82		57		26		6	
Female	4		6		88		27		6	
Age		0.008		<0.001		0.397		0.131		0.001
Youths (14–24)	9		40		51		37		6	
Adults (25–59)	9		67		60		41		9	
Seniors (≥60)	40		37		60		48		48	
Origin		0.019		0.189		0.032		0.002		0.982
Local	14		55		55		44		8	
Non-local	7		58		60		35		13	
Skill level		<0.001		0.048		<0.001		<0.001		<0.001
Beginner	5		11		15		10		10	
Intermediate	4		69		30		25		4	
Expert	18		52		92		60		17	
Total	11		55		13		40		10	

(*) Cited FIS rules (data in %).

**Table 7:** Active knowledge of existing rules (*).

FIS Rules	R6	*p*	R7	*p*	R8	*p*	R9	*p*	R10	*p*
Gender		0.527		0.362		0.277		0.721		0.242
Male	5		2		38		86		6	
Female	4		6		53		88		2	
Age		---		---		<0.001		0.027		---
Youths (14–24)	4		1		9		81		1	
Adults (25–59)	4		4		51		88		4	
Seniors (≥60)	16		16		100		96		40	
Origin		0.129		0.197		0.912		0.003		0.083
Local	6		1		33		84		1	
Non-local	3		7		55		90		10	
Skill level		---		---		<0.001		<0.001		---
Beginner	10		0		0		95		5	
Intermediate	2		0		21		76		3	
Expert	7		8		71		97		7	
Total	5		4		43		86		5	

(*) Cited FIS rules (data in %).

**Table 8:** Passive knowledge of existing rules (*).

Questions	Q1	*p*	Q2	*p*	Q3	*p*	Q4	*p*	Q5	*p*	Q6	*p*
Gender		0.432		0.582		0.493		0.517		0.398		0.417
Male	94		74		77		75		71		91	
Female	96		71		73		75		69		93	
Age		0.090		0.004		0.008		0.003		0.002		0.009
Youths (14–24)	93		67		66		68		62		87	
Adults (25–59)	96		76		80		77		76		95	
Seniors (≥60)	98		91		93		85		76		94	
Origin		0.032		0.585		0.619		0.001		0.971		0.008
Local	88		74		77		81		70		86	
Non-local	99		72		75		69		70		96	
Skill level		<0.001		<0.001		<0.001		<0.001		<0.001		<0.001
Beginner	68		46		54		38		32		49	
Intermediate	97		65		68		67		63		96	
Expert	99		97		97		99		90		99	
Total	95		73		76		74		70		92	

(*) Correct answers in %.

**Table 9:** Passive knowledge of existing rules (*).

Questions	Q7	*p*	Q8	*p*	Q9	*p*	Q10	*p*	Q11	*p*	Q12	*p*
Gender		0.677		0.478		0.182		0.597		0.792		0.046
Male	91		51		43		90		98		42	
Female	95		49		37		93		96		30	
Age		0.047		<0.001		<0.001		0.008		0.072		<0.001
Youths (14–24)	87		15		9		87		95		4	
Adults (25–59)	92		73		61		95		99		58	
Seniors (≥60)	97		83		76		94		98		89	
Origin		0.009		0.667		0.198		0.217		0.982		0.369
Local	87		52		44		86		99		41	
Non-local	96		49		39		95		97		36	
Skill level		<0.001		<0.001		<0.001		<0.001		0.213		<0.001
Beginner	48		19		13		41		83		9	
Intermediate	96		39		27		96		99		22	
Expert	99		81		74		99		99		75	
Total	92		50		41		91		98		38	

(*) Correct answers in %.
